# More about the International Medical Congress and Railroad Rates

**Published:** 1887-08

**Authors:** 


### MORE ABOUT THE INTERNATIONAL CONGRESS AND RAILROAD RATES.

The following letter, from Major Means, general passenger agent Piedmont Air Line, will be read with interest by those who contemplate going to Washington to the International Congress, or for other purposes:

HOUSTON, TEXAS, August 6, 1887.

*F. E. Daniel, M. D.:*

DEAR SIR:—Your's to hand. You can assure the physicians of Texas that there is not a shadow of danger in going through New Orleans. If, perchance, yellow fever *should* prevail in New Orleans, I will have tickets cancelled, and send passengers via some other route. Have sent circulars to all delegates, and will call on most of them next week.

* * I have succeeded in making a round trip rate of one and one-third fare, which will make, from Austin, round trip rate, $53.55; San Antonio, $57.40; Houston, Brenham, Milano, Waco, Fort Worth, Dallas, etc., $51.20. This is much less rate than I expected to have made.

Am making arrangements for through sleepers from Houston to Washington; and will arrange for delegates' comfort at all eating houses. Yours respectfully,

J. M. MEANS.

If the ticket is sold at full fare going, the purchaser must, on returning, procure a certificate that he attended the Congress, in order to entitle him to the one-third rate; but we apprehend it is the intention of the Piedmont Air Line to sell a round trip ticket, good to return within so many days. In that event, no certificate is necessary. Nor is it necessary that one should be a delegate from any Association, in order to become a member of the Congress. We give the above in reply to numerous inquiries we have received from members of the Texas State Association who wish to go *as delegates.*

